# Seq and CLIP through the miRNA world

**DOI:** 10.1186/gb4151

**Published:** 2014-01-25

**Authors:** Nitish Mittal, Mihaela Zavolan

**Affiliations:** 1 Biozentrum, University of Basel, Klingelbergstrasse 50-70, Basel, Switzerland

## Abstract

High-throughput sequencing of RNAs crosslinked to Argonaute proteins reveals not only a multitude of atypical miRNA binding sites but also of miRNA targets with atypical functions, and can be used to infer quantitative models of miRNA-target interaction strength.

## Introduction

In the vast landscape of cellular RNAs of widely different sizes, microRNAs (miRNAs) are small (21 to 22 nucleotides long) RNAs that guide Argonaute proteins to target RNAs to post-transcriptionally regulate their expression [1,2]. lin-4 was the first miRNA to be reported and found to inhibit the translation of the *lin-14* mRNA at a critical stage in the development of the worm *Caenorhabditis elegans*[3,4]. It was the discovery of the evolutionarily conserved let-7 miRNA [5,6], however, that sparked a tremendous interest in RNAs with regulatory functions. Through many studies, a large catalog of miRNAs has since been compiled, from species as evolutionarily distant as viruses and mammals [7]. In the canonical biogenesis pathway, miRNAs are transcribed by the RNA polymerase II (Pol II) as long pri-miRNA. These are processed through two endonucleolytic steps involving RNase III enzymes [8], the first carried out by the Drosha-DiGeorge syndrome critical region 8 (DGCR8) complex in the nucleus to produce pre-miRNAs, and the second by the Dicer-TAR (HIV-1) RNA binding protein 2 (TRBP) complex in the cytoplasm to yield 21 to 22 nucleotide-long double-stranded RNAs. Typically one of the two strands of the duplex is picked up by an Argonaute protein to form a miRNA-guided RNA silencing complex (miRISC). The biogenesis of miRNAs has been reviewed extensively elsewhere [9]. Several alternative miRNA biogenesis pathways have also been described. Mirtrons, for example, bypass Drosha processing, being instead produced from spliced introns by the activity of the lariat debranching enzyme [10]. Another miRNA, pre-miR-451, is not processed by Dicer but rather by the Argonaute 2 (Ago2) protein itself to yield the mature miRNA [11].

Many experimental and computational studies converged on the 5′ end (about nucleotides 1 to 8) of the miRNA (also known as the ‘seed’ region) being generally involved in target recognition through perfect nucleotide complementarity (see [1] for a recent review). Exceptions have also been reported: for example, the let-7 binding site in the *lin-41* 3′ UTR, in which the nucleotide located between those that base-pair with the fourth and fifth miRNA nucleotide is looped out of the miRNA-target hybrid [12,13]. Relatively rare sites that pair with the central region of the miRNA have also been found [14] and the interest in non-canonical miRNA target sites, which do not pair perfectly with the miRNA seed region, persists [15,16]. Putative sites that are computationally predicted to imperfectly pair with the miRNA seed region due to a bulged nucleotide in either the miRNA or the target site are known to exhibit some degree of evolutionary conservation relative to random 3′ UTR fragments of the same length [17,18]. However, the conservation signal as well as the apparent effect of such sites on the stability of the target mRNAs is smaller than those of canonical sites [19]. This likely indicates that only a subset of these sites is functional. Identifying this subset has so far been challenging.

Evolutionary studies of the Piwi-Argonaute-Zwille (PAZ) domain-containing proteins revealed largely two clusters, one corresponding to Argonaute and the other to the Piwi proteins [20]. Members of these clusters appear to have quite exquisite specificities for the length of the small RNAs that they bind [21]. Sequencing of the populations of small RNAs that associate with individual members of this protein family has been recently used to identify not only small guiding RNAs but also their targets. Here we review the insights into the processing of small RNAs and into their biological functions that were derived through high-throughput studies, particularly those that investigated individual protein components of small RNA-containing regulatory pathways.

## High-throughput approaches for identifying small non-coding RNA genes and targets

High-throughput sequencing has revolutionized molecular biology, including the study of RNA. Taking advantage of the biochemical properties of miRNAs (presence of a 5′-phosphate and 3′-hydroxyl), protocols have been developed to isolate and sequence these molecules with very little background [22-24]. The approach consisted of isolation of total RNA, followed by separation on urea-containing 15% polyacrylamide gel along with a ^32^P-labelled ladder to allow identification of RNAs of the appropriate size. After cutting the corresponding band out of the gel and elution of the RNA overnight, 3′ and 5′ adaptors were ligated, the fragments concatamerized, and cDNA synthesized, PCR-amplified, cloned into plasmid vectors and sequenced with the Sanger method to yield 100 to 1,000 small RNAs per sample. Next generation sequencing (NGS) greatly increased the yield to 10^4^ to 10^5^ small RNA sequences per sample in the initial studies employing this technology [25-27]. NGS-based approaches have since been used to identify many other types of small RNAs. The basic protocol remains largely the same, except that cDNAs are sequenced without cloning and concatamerization [28].

**Figure 1:**
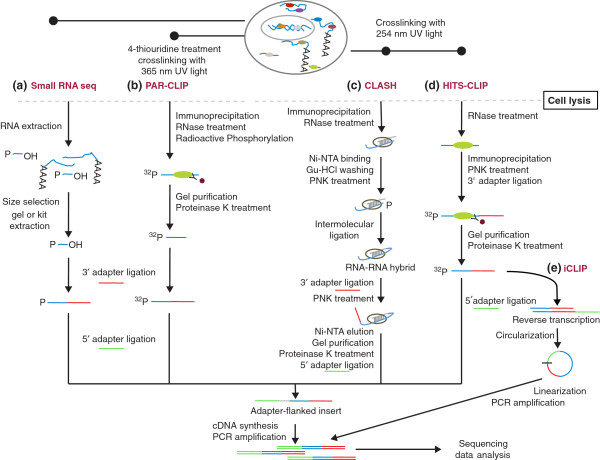
**High-throughput methods for sequencing small RNAs and their targets.** Conceptual protocols highlighting the differences between the methods for deep sequencing of **(a)** small RNAs and of **(b-e)** small RNA targets (PAR-CLIP **(b)**, CLASH **(c)**, HITS-CLIP **(d)**, iCLIP **(e)**). Ni-NTA, nickel nitrilotriacetic acid; Gu-HCL, guanidine hydrochloride; PNK, polynucleotide kinase.

To further remove the background of processing products of abundant cellular RNAs as well as to gain more direct insight into the functions of small RNAs, protocols that employ the pulldown of the protein of interest with a specific antibody have also been proposed (Figure 1). They have been used in the discovery of miRNAs and various other non-coding RNAs that associate with Argonaute proteins [29,30]. Building on this approach, the Darnell group [31,32] further applied a step of *in vivo* crosslinking using ultraviolet (UV) C light (254 nm) of the RNA-binding protein (RBP) to the RNAs with which it interacts in intact cells or tissues. After cell lysis, the RNA is partially digested to yield fragments in the range of 30 to 50 nucleotides, the RNA-protein complex is immunoprecipitated with an antibody specific to the protein of interest, the RNA in the complex is radioactively labeled at the 5′ end with ^32^P, and an adapter is ligated at the 3′ end, after which the RNA-protein complex is separated on an SDS gel and transferred to a nitrocellulose membrane. This step results in the removal of unbound RNAs and retention of the covalently crosslinked RNA-protein complex. After the protein is digested from the complex with proteinase K, a 5′ adapter is ligated, cDNA is synthesized and PCR amplification is carried out with primers complementary to 3′ and 5′ adapters. The PCR adapters also carry sequences needed for attachment to the flowcell surface and for attachment of the sequencing primers, when sequencing on Illumina platforms. The resulting library is subjected to NGS. To further improve the efficiency of capture of miRNA targets, the Tuschl group proposed a modified protocol, photoactivatable ribonucleoside-enhanced crosslinking and immunoprecipitation (PAR-CLIP), in which photoactivatable ribonucleoside analogues such as 4-thiouridine (4-SU) or 6-thioguanosine (6-SG) are incorporated into RNAs before crosslinking [33]. These modified nucleotides can be efficiently crosslinked to proteins using UV A (365 nm). In addition, crosslinking-diagnostic mutations (T-to-C or G-to-A, respectively) are introduced during reverse transcription to allow determination of the binding sites at close-to-nucleotide resolution. This protocol has been successfully used to identify not only miRNA targets [33,34] but also the RNA targets of many RNA-binding proteins [35]. To achieve the desired single-nucleotide resolution in the identification of RBP targets, a method that takes advantage of the propensity of reverse transcriptase to stop at the position of crosslinking has been proposed [36]. This individual nucleotide resolution CLIP method (iCLIP) has only very recently been applied to the characterization of small RNA-guided interactions [37].

**Figure 2:**
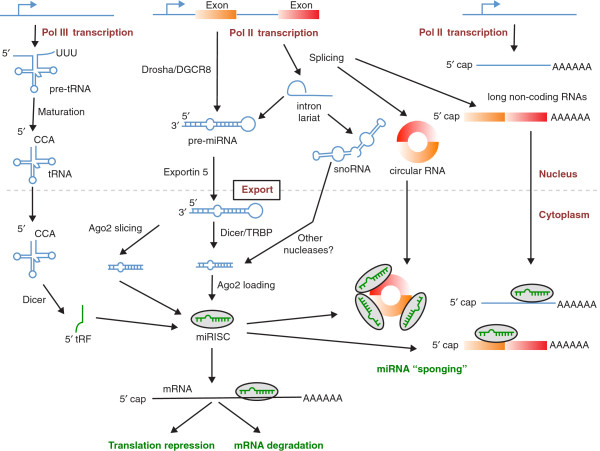
**The multi-faceted miRNA biogenesis and miRNA interaction with targets.** miRNAs are processed mainly by Drosha-DGCR8 in the canonical pathway, but also by the lariate-debranching enzyme in the nucleus, and by Dicer (from other non-coding RNAs such as tRNAs and snoRNAs) and Ago2 in the cytoplasm. Although miRISC generally regulates the stability and translation rate of target mRNAs, other long RNAs feed back on the miRNA regulation by sequestering miRNAs from their direct targets.

Although high-throughput sequencing of RNA isolated by crosslinking immunoprecipitation (HITS-CLIP), PAR-CLIP and iCLIP have a similar basis, their differences make them more or less applicable in specific contexts. For instance, an important advantage of HITS-CLIP is that it can be performed with relative ease in both cultured cells and living tissues. However, the efficiency of crosslinking of Argonaute to the mRNA targets (as opposed to the guide RNAs) appears lower than with PAR-CLIP. Although PAR-CLIP is more difficult to carry out in tissues, its successful application to the identification of *in vivo germline development defective 1* (GLD-1) protein binding sites in the worm *C. elegans* has been reported [38]. Important concerns about the use of photoreactive nucleosides are that they are toxic for cells [39] and they bias the set of binding sites that can be identified. However, the concentration of 4-thiouridine that has been used in PAR-CLIP experiments has not been found to obviously affect the cells [33]. On the other hand, the bias in binding site identification remains largely unquantified. Yet this is not only an issue for PAR-CLIP because crosslinking with 254 nm UV, as in HITS-CLIP, also targets uridines preferentially [40].

Generally, it has become clear that crosslinking-induced mutations are useful in separating the signal from noise and identification of high-affinity binding sites [34,40,41], but how different CLIP methods compare in this regard needs to be further investigated. Several factors make this comparison difficult. First, the protocols are lengthy and difficult to master, which makes it difficult to obtain equally good data with all the different CLIP protocols. Second, the possible interplay between the biases of individual approaches and the sequence specificity of individual proteins makes it necessary to perform the comparison on multiple proteins. Third, it is non-trivial to obtain independent quantifications of occupancies of individual binding sites by a given protein, which is necessary for evaluating the results of different CLIP protocols. One possibility is to use an *in vitro*-derived model of the sequence specificity of the protein to predict its affinity for individual CLIPed sites [34]. The success of this approach depends on how accurately the affinity of RBP-RNA interactions can be predicted. Another approach would be to take advantage of proteins that establish crosslinks to RNA in a UV-independent manner. For example, the NOP2/Sun domain family, member 2 protein (NSUN2) normally catalyzes methylation of cytosine to 5-methylcytosine, generating a protein-RNA crosslink as an intermediate in the process. Employing a variant that can no longer resolve the covalent bond that the protein forms with the RNA, the binding sites of this protein could be determined without UV crosslinking and compared with the binding sites obtained by crosslinking the protein to its sites with UV light. Finally, in the absence of independent measures of site occupancy, comparisons of sequence biases around putative binding sites inferred for different proteins have been performed [40]. They indicate that UVC light preferentially induces crosslinking of uridines. Furthermore, it appears that reverse transcriptase stoppage sites that are captured through iCLIP are a more accurate indicator of protein binding sites than nucleotide deletions that are introduced during HITS-CLIP.

Although the above-mentioned methods are able to identify the endogenous targets of miRNAs or other small non-coding RNAs, they do not directly reveal which small RNA guided the interaction of the RBP with individual targets. To address this issue, another experimental approach has been very recently proposed. It is known as crosslinking, ligation and sequencing of hybrids (CLASH) and it relies on the ligation of the guide RNA to the target RNA within the ternary guide RNA-target RNA-RBP complex, after the immunoprecipitation of the protein with the bound RNAs [42]. In contrast to CLIP, this protocol includes, after immunoprecipitation and partial digestion of the RNA in the RNA-protein complex, a purification step based on a 6x-histidine epitope tag that allows denaturing purification of the RNA-protein complex on nickel beads at 6 M guanidine-HCl. This ensures that only the RNA that is covalently linked to protein is purified. In addition, an inter-molecular RNA-RNA ligation step is introduced to capture the target site and the miRNA from the RNA-protein ternary complex. After elution of the RNA-protein complex from nickel beads, the sample preparation proceeds similarly to CLIP. This method has been successfully used to identify various types of RNA-RNA hybrids [43], and its recent application to the Ago1 protein led to the suggestion that different miRNAs may have different modes of binding to their target mRNAs [42]. In its current form, CLASH has very low efficiency, with only about 2% of the reads obtained in an experiment corresponding to miRNA-target hybrids. Furthermore, the use of a 6x-histidine tag for the purification of RNA-protein complexes makes the protocol applicable only to cells that express the tagged protein.

## The expanding set of miRNA targets

Following the model of worm miRNAs, initial large-scale studies of miRNA targets focused on mRNAs, first attempting to predict them computationally [44-46] and then to determine them experimentally, by virtue of the change in their expression upon miRNA transfection measured with microarrays [47]. More recently, crosslinking-based approaches are starting to bring a new understanding of miRNA-target interactions and to uncover unusual targets (Figure 2).

### Identification of non-canonical miRNA target sites from CLIP data

miRNA target sites that do not perfectly pair with the miRNA seed region (so-called non-canonical sites) have been both described experimentally [5,12,15,48] and predicted based on evolutionary conservation [49]. However, recent analyses of Ago2-CLIP data underscored the relative abundance of a specific type of site, in which the nucleotide located between those that pair with positions 5 and 6 of the miRNA is looped out in the target [16,50]. More importantly, CLIP provided sufficient data to infer a biophysical model of miRNA-target site interaction [19] that allows, for the first time, a quantitative evaluation of the strength of canonical and non-canonical interactions. As a result, functional non-canonical target sites could be identified with high accuracy. They amounted to approximately a quarter of the high-confidence, reproducibly CLIPed sites. Perhaps as expected, abundant miRNAs were found to have a higher proportion of non-canonical sites compared with the less expressed miRNAs. A recent study that captured and sequenced miRNA-target site pairs [42] suggested that miRNAs differ widely in their propensity to engage in non-canonical modes of interaction with their targets. miR-92a, for example, a member of the abundantly expressed miR-17/92 cluster of miRNAs, appeared to predominantly pair with targets through its 3′ end region. The response of these targets to the miR-92a depletion was, however, smaller than that of seed-type miR-92a targets, and thus the significance of these non-canonical interactions remains to be determined. Nonetheless, as more CLASH datasets emerge, it will be interesting to apply the MIRZA inference procedure described in Khorshid *et al*. [19] to CLASH data to infer miRNA-specific modes of interaction with the targets. The MIRZA approach can be further adapted to infer miRNA-target interaction parameters from measurements of interaction affinity [51]. A comparative analysis of models inferred from *in vivo* and *in vitro* data should ultimately reveal the properties of functionally relevant miRNA target sites.

### Long non-coding RNA targets and miRNA sponges

Although the vast majority of Ago2 targets are mRNAs, a variety of non-coding RNA targets have also been identified. For example, about 5% of the Ago2 targets obtained in HITS-CLIP samples from mouse brain were long non-coding RNAs (lncRNAs) [32], and many lncRNA-miRNA interactions were also inferred from PAR-CLIP data of different Argonaute proteins [52]. lncRNA-Argonaute interactions (for example, between *XIST* lncRNA and hsa-miR-370-3p) are documented in the starBase database [53]. Rapidly emerging evidence points to a function of lncRNA-miRNA interactions in regulating the availability of the miRNA itself, with the lncRNA functioning as a miRNA sponge.

miRNA sponges were introduced a few years ago [54] as competitive miRNA inhibitors consisting of transgenic RNAs that contain multiple putative binding sites for a given miRNA or miRNA family. Perhaps not surprisingly, natural miRNA sponges have emerged as well, initially among viral transcripts. For example, a U-rich RNA of the *Herpesvirus saimiri* acts as a sponge for the host miR-27 [55], as does the m169 transcript of the murine cytomegalic virus [56]. In mammals, pseudogenes such as *PTENP1* and *KRASP1*[57] have been proposed to sponge miRNAs that would otherwise act on the corresponding genes. It remains unclear, however, whether under normal or disease conditions these pseudogenes are expressed at sufficient levels to be effective as sponges [58]. Other lncRNAs do appear to accumulate at very high levels, consistent with a sponging function. For example, a very recent study showed that the lncRNA *H19* associates with the RISC complex, sequestering the let-7 miRNA and thereby modulating the expression of let-7 targets [59]. A similar interaction has been proposed to occur between lincRNA-RoR and miR-145 [60].

### Circular RNA

miRNA sponges have also been found among circular RNAs (circRNAs). Although a few circRNAs, such as those derived from the *DCC* tumor suppressor gene [61], the testis-determining *SRY* gene [62], *ETS-1*[63] and the cytochrome P450 gene *2C24*[64], were described two decades ago, it was thought that such RNAs are rare, aberrant products of the splicing reaction [61,63]. Deep sequencing of RNAs from a variety of normal and malignant cells revealed, however, an abundance of such transcripts [65,66] that can be expressed at 10-fold higher levels than the mRNAs derived from the corresponding genes [67]. The biogenesis of circRNA is not yet clear. Models such as lariat-driven or intron-pairing-driven circularization have been proposed [67]. Furthermore, failure in debranching can also yield intron-derived circRNAs [68]. Interestingly, Ago2-PAR-CLIP revealed that a circRNA that is antisense to the cerebellar degeneration-related protein 1 transcript (*CDR1as*) is densely bound by Argonaute proteins, guided by a large number of conserved miR-7 binding sites [69]. The circRNA is completely resistant to miRNA-mediated target destabilization and it strongly suppresses miR-7 activity in the mouse and zebrafish brain [69,70]. Other functions of circRNAs, such as in Pol II-dependent transcription, have also been reported [68].

The adoption of high-throughput approaches is not without complications. Every method has limited accuracy and even in deep sequencing samples one expects a certain amount of contaminating RNAs, particularly originating from abundant cellular RNAs. Although *a priori* knowledge of abundant RNA species generally helps in sifting away this background, novel variants of well-studied molecules, such as tRNA-derived fragments (tRFs) and small nucleolar RNAs (snoRNAs), have also been identified recently, complicating the analysis of deep-sequencing datasets. We will describe here some non-canonically processed RNAs with biological significance, whose number appears to be more limited than initial analyses suggested [71-74].

### Remodeling of the miRNA targetome upon stress

Application of Ago2-CLIP revealed a stress-dependent remodeling of miRNA-target interactions, canonical interactions becoming more prominent upon arsenite stress [75]. Increased Ago2 binding to these canonical sites was also associated with increased repression. The mechanism behind the redistribution of Ago2 binding to higher affinity, canonical sites under stress remains to be identified. The abundance of both miRNAs and Ago2 protein appears to remain unchanged between conditions and it was rather proposed that signal-induced post-translational modifications of Ago2 may alter the interaction strength at specific sites. It is conceivable that a reduction in RISC affinity for target sites leads to reduced binding to weak, non-canonical sites. However, changes in the overall abundance of miRNA target sites may also lead to changes in the stringency of competition for a limited number of RISC complexes, and to a redistribution of Ago2 between low- and high-affinity sites.

## More roads leading to RISC

### IsomiRs

Although mature miRNAs are typically processed very precisely from their precursor molecules, evidence is accumulating that some miRNA variants - isomiRs - that differ in a few nucleotides from the canonical, most frequently observed sequence are generated and have biological significance. Some isomiRs are templated, being the result of imprecise cropping of miRNA precursors by Drosha or Dicer [76] or of the trimming of the miRNA 3′ end by 3′-to-5′ exoribonucleases such as Nibbler in *Drosophila*[77] and QIP in *Neurospora*[78]. The Dicer partner TRBP can also modulate isomiR generation [79,80]. When the miRNA is encoded in the 3′ arm of the pre-miRNA, the Dicer-modulated change in isomiR abundance will likely lead to a change in the spectrum of mRNAs that are targeted by the miRNA. For example, the 5′ isomirs of mir-307a do seem to have distinct targets because the glycerol kinase and taranis mRNAs are repressed by mir-307a^23-mer^ but not by mir-307a^21-mer^[80]. Furthermore, isomiRs and their canonical counterparts appear to associate equally with polysomal, translated RNA [81], indicating that they may indeed function as miRNAs. A variety of terminal nucleotidyl transferases, such as mitochondrial poly(A) polymerase (MTPAP), PAP associated domain containing (PAPD)4, PAPD5, zinc finger, CCHC domain containing (ZCCHC)6, ZCCHC11 and terminal uridylyl transferase 1, U6 snRNA-specific (TUT1) [82], have been implicated in the generation of non-templated 3′ isomiRs. TUT1-dependent addition of terminal U nucleotides has been implicated in the regulation of miRNA stability [83].

### snoRNA-derived small RNAs and tRFs

Sequencing of small RNA populations, including those that specifically associate with RISC proteins, revealed fragments derived from abundantly expressed structural RNAs, such as snoRNAs and tRNAs, that also seem to associate with Argonaute proteins [29,84]. Among the snoRNAs, the H/ACA box-type, which forms a typical two-hairpin structure, gives rise to miRNA-like molecules that amount to a few percent of the Argonaute-associated small RNA population [84]. The H/ACA box snoRNA small Cajal body-specific RNA 15 (SCARNA15) generates the most abundant Ago2-associated snoRNA-derived small RNA, which targets the transcript encoding the Mediator coactivator complex subunit cyclin-dependent kinase 19 (CDK19) [29]. Although less abundant among the approximately 20 to 40 nucleotide-long RNAs in the cell, tRFs appear to associate more efficiently with the Ago2 protein compared with snoRNA-derived fragments [84]. Various nucleases have been implicated in the generation of tRFs, starting with Dicer, which processes the CU1276 tRF - which functions as a miRNA in B cells, repressing the replication protein A1 [85] - and the tRF-5-GlnCTG [86]. Angiogenin acts at the TψC loop to generate 3′-end tRFs, and on the anticodon loop to produce 5′-end tRFs [87]. The latter have been implicated in the eukaryotic translation initiation factor 2 alpha (eIF2α)-independent inhibition of translation in U2OS cells upon stress [88]. Finally, the elaC ribonuclease Z 2 (ELAC2) endonuclease cleaves the 3′ trailer sequence from Ser-TGA pre-tRNAs, generating the pro-proliferative trf-1001 tRF [89].

## Cleaving without a guide

Although we have extensively discussed small RNA-guided mRNA destabilization, the Drosha-DGCR8 complex that processes pri-miRNAs also cleaves hairpin structures that form within other molecules, including mRNAs, thereby inducing their destabilization. The abundance of the metastasis associated lung adenocarcinoma transcript 1 (non-protein coding) (*MALAT1*) non-coding RNA appears to be controlled through this mechanism [90], as is the expression of several genes that induce neuronal differentiation, such as neurogenin 2 [91].

## Conclusions

The list of long and short functional RNAs is expanding rapidly. Here we have summarized some of the insights into the targets of the miRNA-dependent pathway that were obtained particularly though NGS-based approaches such as small RNA sequencing and different variants of RBP-CLIP methods. An increasing number of entry points into miRNA-dependent gene regulation are being discovered. Furthermore, miRNA-target interactions are plastic, and cell type- and condition-dependent. Nonetheless, quantitative analyses in the context of computational models should ultimately allow the behavior of this very complex gene regulatory system to be understood and predicted.

## Abbreviations

Ago: Argonaute; circMRNA: Circular miRNA; CLASH: Crosslinking ligation and sequencing of hybrids; DGCR8: DiGeorge critical region 8; HITS-CLIP: High-throughput sequencing of RNA isolated by crosslinking immunoprecipitation; iCLIP: Individual nucleotide resolution CLIP method; lncRNA: Long noncoding RNA; miRISC: miRNA-guided RNA silencing complex; miRNA: microRNA; NGS: Next generation sequencing; PAR-CLIP: Photoactivatable ribonucleotide-enhanced crosslinking and immunoprecipitation; PCR: Polymerase chain reaction; Pol II: RNA polymerase II; RBP: RNA-binding protein; RISC: RNA silencing complex; snoRNA: Small nucleolar RNA; TRBP: TAR (HIV-1) RNA binding protein 2; tRF: tRNA-derived RNA fragments; tRNA: Transfer RNA; UTR: Untranslated region.
